# Using autogenous tooth sticky bone graft repair mandibular third molar dentigerous cyst osseous defects

**DOI:** 10.1186/s12903-023-03673-w

**Published:** 2024-01-07

**Authors:** Alimujiang Wushou, Yuan Luo, Qing-tao Cheng, Zhi-cheng Yang

**Affiliations:** 1 Department of Oral & Maxillofacial Surgery, Shanghai Stomatological Hospital & School of Stomatology, Fudan University, Shanghai, China; 2 Shanghai Key Laboratory of Craniomaxillofacial Development and Diseases, Fudan University, Shanghai, China

**Keywords:** Dentigerous cyst, Impacted third molars, Autogenous tooth sticky bone graft

## Abstract

**Background:**

Dentigerous cyst are most common odontogenic cyst and they frequently occur at the mandibular third molar. Their asymptomatic long medical history always resulted in severe bone resorption at the distal aspect of the adjacent second molar. BonMaker® ATB demonstrate an excellent autogenous bone graft candidacy. The aim of this study is to share a single team’s experience of dentigerous cyst osseous defect repairing by applying autogenous tooth sticky bone graft.

**Method:**

In total, 18 patients with dentigerous cyst, which was arised from mandibular third molar unilaterally, were enrolled in this study. Enucleation of dentigerous cyst was performed extracting with involving teeth under general anesthesia. Autogenous tooth sticky bone graft was prepared using extracted tooth and autogenous fibrin glue. Subsequently, grafting was performed above covering with concentrate growth factors. Patients were followed up at sixth months.

**Results:**

They were eleven male and seven female patients. Their ages ranged from 20 to 40 years, with a mean of 31 years. Primary wound healing of all sites was achieved in all the patients. Sixth months postoperative radiographic assessment show that dentigerous cysts osseous defects of seventeen patients were good bone filling and ossification. One patient occurred slight bone resorption at the distal aspect of the adjacent second molar.

**Conclusion:**

Within the limitation of sample size and retrospective nature of the present study, autogenous tooth sticky bone graft demonstrates one of the best alternative alveolar bones repairing graft.

## Introduction

Jaw cysts are the most common oral diseases. Based on the histologic origin, they are divided into odontogenic cysts and nonodontogenic cysts [1]. Dentigerous cysts, also called follicular cysts, are odontogenic cysts and are the second most frequently encountered lesions clinically. According to previously published data on the prevalence of dentigerous cysts, patient ages range from 6–99 years, with an average of 35 years, and the peak incidence centers in the second and third decades of life, with a male predilection. Approximately 80% of dentigerous cysts occur at the mandibular third molar, approximately 10% are located at the maxillary canine, and the remaining rarely involve the rest of the teeth [2]. The clinical manifestations of dentigerous cysts are often asymptomatic. Most of them are found during impacted third molar radiographic examination. However, a few cases presented slow-growing palpable masses with a long medical history and resulted in slight asymmetrical facial deformity and bone resorption [3].

After diagnosis, the treatment procedure will be carried out depending on the expansion size of the cyst. The main treatment modalities include enucleation of the cyst plus extraction of the involved teeth and marsupialization [4]. Although the prognosis is good, both approaches have disadvantages. For example, marsupialization of third molar dentigerous cysts requires a long treatment period, and a second surgery is often needed. Enucleation plus extraction of the involved teeth often results in bone defects [5]. Generally, the longer the medical history, the more severe the bone resorption around the crown of the mandibular third molar region. Extensive bone resorption often affects the adjacent teeth. The most common condition is loosening of the adjacent teeth due to distal alveolar bone resorption. Spontaneous bone defect healing without restoration will further jeopardize the condition of the adjacent teeth. Marsupialization with or without extracting the involved teeth could also not improve the condition of the adjacent teeth [6].

For dentigerous cyst bone defects, autogenous bone grafting (ABG) and guided bone regeneration (GBR) are the mainstream bone reconstruction modalities [7]. ABG is the gold standard of alveolar bone reconstruction and the first choice for complex repair. However, its “rob Peter to pay Paul” supplying patterns resulted in high bone resorption and complex donor site postoperative complications [8]. There is accumulating evidence for using autogenous teeth as a grafting material for alveolar bone restoration, socket preservation, and maxillary sinus lifting, and this method shows excellent osteoconductivity, osteoinductivity and osteogenicity [9, 10]. There have been no studies that have described the repair of bone defects of dentigerous cysts arising from mandibular third molar grafts prepared with autogenous tooth graft material [11]. In this study, we present our experience in repairing dentigerous cysts arising from mandibular third molar bone defects by using autogenous tooth sticky bone graft (ATSBG) from extracted third molars with or without other residual crowns and roots that need to be extracted at the same time.

## Materials & methods

### Patients

From January 2021 to January 2023, 18 consecutive patients with dentigerous cysts arising from mandibular impacted third molars and reconstructed with ATSBG prepared from extracted third molars with or without other residual crowns and roots that needed to be extracted at the same time at the Department of Oral & Maxillofacial Surgery, Shanghai Stomatological Hospital & School of Stomatology, Fudan University were retrospectively studied with their clinical data. The study patients’ inclusion criteria were as follows: (1) a pathologically confirmed dentigerous cyst; (2) a dentigerous cyst arising from an impacted third molar and total proximal alveolar bone loss; (3) at least two impacted mandibular third molars with or without other residual crowns and roots that needed to be extracted at the same time with dentigerous cyst surgery; (4) no contraindications to tooth extraction under general anesthesia; (5) age of 20 ~ 40 years with good oral hygiene; (6) no history of drinking alcohol and smoking; and (7) no history of any other drug use. The exclusion criteria were as follows: (1) adjacent second molar with periapical inflammation, crowding, ectasia, and torsion; (2) pregnant or breastfeeding; (3) dentigerous cyst arising the third molar with severe pericoronitis that has not been effectively controlled; (4) multiple dentigerous cysts arising from mandibular third molars. The relevant contents of the study were extracted from the inpatient records. Preoperatively, all patients signed an informed consent form. Preoperative and postoperative panoramic radiographs and cone-beam computed tomography were retrieved from the image database. This study was approved by the institutional review board.

### Surgical procedure

The whole surgical procedure was carried out under general anesthesia by the listed authors. First, enucleation of the dentigerous cyst was performed. A modified triangular flap was used uniformly, and a full-thickness mucoperiosteal flap was flipped to expose the impacted third molar. Minimally invasive extraction of the impacted third molar, enucleation and adjacent second molar root planing were performed consecutively. As long as the patients had indications for removal of other residual crowns and roots, they were extracted at the same time with dentigerous cyst surgery. In order to reduce bone loss in the whole operation, piezoelectric and sonic instruments were used in combination.

**Fig. 1:**
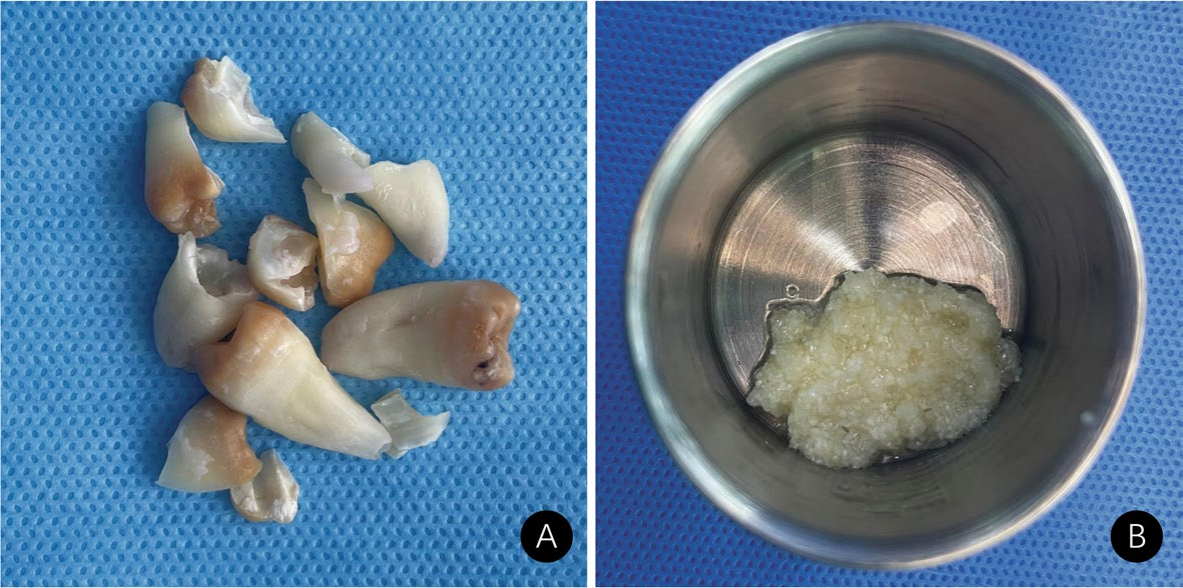
**A** Extracted tooth for preparation of autogenous tooth bone graft power. **B** Autogenous tooth sticky bone graft

Second, after the extracted tooth was washed with saline and hydrogen peroxide and dried, an autogenous tooth graft was prepared chairside using a Bonmaker® device (http://www.bonmaker.net/) following the developers’ instructions (Fig. 1A). Three tubes of venous blood were drawn for every 10 ml from every patient. Subsequently, one tube of autogenous fibrin glue and two tubes of concentrated growth factor (CGF) were prepared according to the manufacturer’s instructions (http://www.trausim.com). The autogenous tooth graft powder was placed in specific round metal storage boxes, and autogenous fibrin glue, after being extracted with a syringe, was added to the autogenous tooth graft powder. After 15 min of agglutination, the gelatinous ATSBG was ready for implantation (Fig. 1B).

**Fig. 2:**
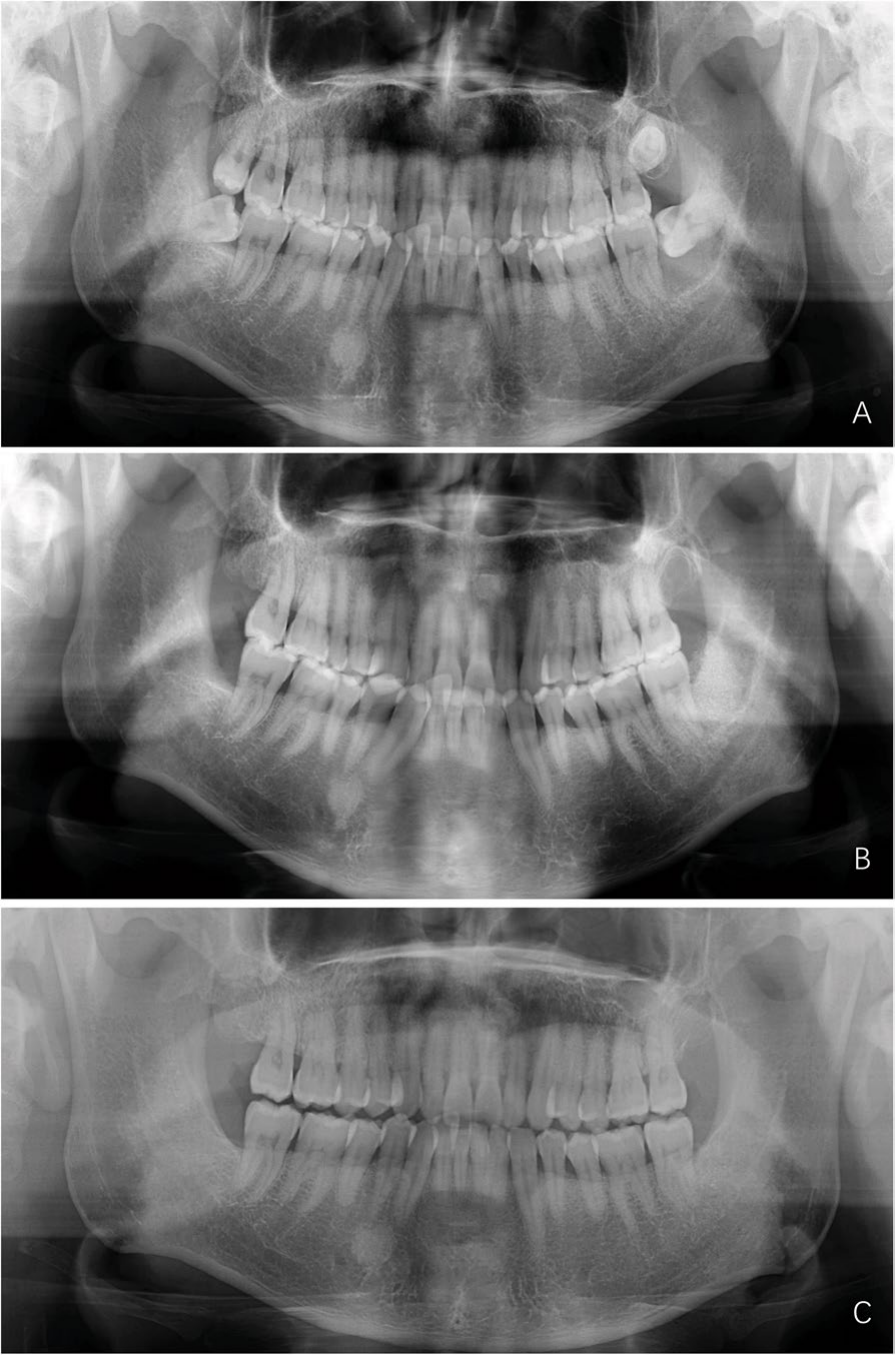
Preoperative and postoperative comparison of left third molar dentigerous cysts and repair: **A** Preoperative panoramic radiographs, **B** One-week postoperative panoramic radiographs, **C** Sixth month postoperative panoramic radiographs

Finally, before grafting, the dentigerous cyst bone defects were bathed with dilute iodophor, hydrogen peroxide and saline solution consecutively. Then, the freshly prepared ATSBG were implanted in layers into the osseous defects. As long as the bottom of the defects was too close to the inferior alveolar neural tube, collagen sponges were placed above to buffer. After the placement of a CGF membrane on the surface, the mucoperiosteal flap was repositioned and closed with mattress plus interrupted sutures. According to the perioperative medication of dental grafting, the application of antibiotics, prednisone and icing was prescribed according to our previous investigation [12]. Suture removal was scheduled one week after surgery. A postoperative radiographic osteogenic evaluation was performed after six months (Fig. 2A, B, C).

## Results

In total, 18 patients with dentigerous cysts in the mandibular region who fulfilled all the inclusion criteria were selected. The cohort included eleven men and seven women. Their ages ranged from 20 to 40 years, with a mean of 31 years. Four patients had residual crowns and roots except the third molar that were extracted at the same time as dentigerous cyst surgery. Primary wound healing of all sites was achieved in all the patients without other complications except in one patient, who had three weeks of transient lower lip numbness, and methylcobalamin tablets were prescribed. Four patients had postoperative adjacent second molar loosening, which returned to normal six months after surgery. The six months postoperative radiographic assessments showed that the dentigerous cyst bone defects in seventeen patients had good bone filling and ossification. Only one patient experienced gingival atrophy because of poor local hygiene, and slight bone resorption occurred distal to the second molar. The baseline clinical characteristics of the enrolled patients are summarized in Table 1.

**Table 1:** Baseline characteristics of included patients

**Parameters**	*n* = 18
**Age (years)**	
Mean	31
Range	20–40
**Gender**	
Male	11
Female	7
**Location of the lesion**	
Right	8
Left	10
**Proximity to the mandibular canal**	
Yes	4
No	14
**Residual crown and root except third molar**	
Presence	4
Absence	14
**Pocket depth of second molar distal aspect (mm)**	
6-month	3.2 ± 1.0

## Discussion

Dentigerous cysts, alveolar bone defects and restoration, and autogenous tooth grafts are not new topics. In this investigation, for the first time, we report unilateral mandibular third dentigerous cyst bone defect reconstruction by applying sticky bone graft, which was prepared with an extracted third molar with or without other residual crowns and roots that were extracted at the same time as dentigerous cyst surgery. ABG, GBR, platelet-rich fibrin (PRF) combined with various bone graft material are the most commonly used alveolar bone repairing approaches [13, 14]. Compared to these methods, ATSBG plus CGF coverage demonstrates the following advantages: first, low inflammatory response and fast soft tissue healing; second, fast bone formation; and last but not least, very low bone resorption rate.

In dentigerous cyst bone defect repair, we have followed the “PASS” principles (P: primary wound coverage without tension; A: angiogenesis, adequate blood supply; S: space, creation/maintenance; S: stability of the wound) [15]. ATSBG are special because of their direct bone formation and low bone resorption; therefore, unlike general GBR, periosteal covering and fixation are generally not needed. Instead, we used bilayer CGF for graft coverage. It has been confirmed that piezosurgery and CGF plays an important role in reducing the inflammatory response and promoting soft tissue healing [16, 17]. Generally, triangular mucoperiosteal flaps are used for mandibular impacted third molar extraction [18]. In this investigation, we used a modified triangular mucoperiosteal flap, and the incision extended forward to the mesial side of the second molar. The benefit of this design is that one was to better expose the lesion and third molar, and the other was to achieve tension-free wound closure.

The limitations of this investigation and the grafting material itself should be acknowledged. “Demand exceeding supply” is the primary sore point in autogenous tooth bone grafting [9–11, 13, 19–21]. In this investigation, this issue was also encountered. Nearly one-fifth of dentigerous cyst bone defects cannot be fully filled with ATSBG. Under these circumstances, the bottom and distal sides of the defect were filled with a collagen sponge. On the one hand, collagen sponges act as a buffer; on the other hand, ATSBG were mainly placed at the distal osseous defects of the second molar and periodontal condition of all the study population were markedly improved. It has a good application prospect in periodontal alveolar bone repairing. Preparation of ATSBG is time-consuming and takes approximately 25–30 min to prepare. It is not fully automated and includes hand tooth cleaning, drying, grounding, and final Bonmaker® chemical treatment [22].

## Conclusion

Over the past several decades, extensive studies have investigated various alveolar bone repair materials, including allograft bone, synthetic bone, and tissue-engineered bone. Although ABG has been dominant in alveolar bone restoration for a long time, it still is not an ideal graft material terminator. Growing evidence has confirmed tooth bone grafts as the desired graft material, and our study results are new evidence. It should be acknowledged that because of the “supply and demand” barrier, large osseous defects cannot be repaired with autogenous tooth bone grafts. For this barrier, allogeneic tooth graft probably answer in the future. Another highlight of the current investigation is that this is the first report of ATSBG and the largest osseous defect of jaw bone repair with ATSBG.

## Data Availability

All data generated or analyzed during this study are included in this published article.
